# Reviving weight loss and metabolic obesity-related comorbidities: mid-term results of single anastomosis sleeve ileal (SASI) bypass for failed sleeve gastrectomy. A retrospective cohort study

**DOI:** 10.1097/JS9.0000000000002874

**Published:** 2025-07-01

**Authors:** Sergio Carandina, Silvia Ferro, Massimiliano DE Palma, Andrea Sartori, Viola Zulian, Antonio Iannelli

**Affiliations:** a ELSAN, Clinique Saint Michel, Centre de Chirurgie de l’Obésité (CCO), Toulon, France; b Department of Digestive and Bariatric Surgery, Clinica Madonna della Salute, Porto Viro, Italy; c Adipocible Research Study Group (Université Nice Côte d’Azur and Initiative d’Excellence – Idex) – Nice, France University of Nice Côte d’Azur – Nice, d’Azur – Nice, France

**Keywords:** bariatric surgery, bypass, failed sleeve gastrectomy, metabolic obesity-related comorbidities, metabolic surgery, single anastomosis sleeve ileal (SASI), sleeve gastrectomy failure, surgical revision

## Abstract

**Background:**

The single anastomosis sleeve ileal (SASI) bypass, a hybrid bariatric procedure combining sleeve gastrectomy (SG) and ileal bypass, has emerged as a potential solution for SG failure, offering both restrictive and malabsorptive effects. This study investigates the mid-term outcomes of SASI in patients undergoing conversion due to SG failure, with a focus on weight loss, metabolic improvement, and postoperative complications.

**Methods:**

This retrospective study included 44 consecutive patients who underwent SASI after SG failure at a tertiary bariatric referral center between May 2019 and June 2024. Patients were assessed for demographic, anthropometric, and comorbidity data, with follow-up at 6, 12, 18, and 24 months. Primary outcomes included weight loss and improvement in comorbidities, while secondary outcomes focused on surgical complications and procedure-related issues.

**Results:**

The mean BMI decreased from 39.1 ± 7.2 kg/m^2^ at the time of SASI to 30.5 ± 5.9 kg/m^2^ and 27.5 ± 4.8 kg/m^2^ at 12 and 24 months, respectively, with a %TWL of 21.5 ± 7.8% at 12 months and 29.7 ± 9.5% at 24 months. Significant improvements were observed in obesity-related comorbidities, including remission of type 2 diabetes, sleep apnea, and hypertension. Short-term complications occurred in 11.3% of patients, with no postoperative mortality. Mid-term follow-up revealed that 65% of patients experienced resolution of gastroesophageal reflux disease (GERD), although 11.3% developed de novo GERD symptoms.

**Conclusion:**

SASI conversion after SG failure is associated with significant weight loss and favorable metabolic outcomes. However, GERD remains a challenge, and careful patient selection and surgical technique are crucial. Larger, multi-center studies with longer follow-up are needed to further refine the role of SASI in revisional bariatric surgery.

## Introduction

The single anastomosis sleeve ileal (SASI) bypass is a hybrid bariatric procedure that combines a restrictive component, sleeve gastrectomy (SG), with an intestinal bypass of variable length between the stomach and the small intestine. This approach integrates SG and ileal bypass elements, offering both restrictive and malabsorptive effects. Initially developed as a single-step, hybrid procedure to address obesity and its associated metabolic disorders, SASI has gained popularity as an option for revisional surgery in patients experiencing failure following SG, similar to the historical evolution of the duodenal switch^[1^-^3]^.

**HIGHLIGHTS**

- The single anastomosis sleeve ileal (SASI) bypass has emerged as a potential solution for SG failure, offering both restrictive and malabsorptive effects.
- This study investigates the mid-term outcomes of SASI in patients undergoing conversion due to SG failure
- This retrospective study included 44 consecutive patients who underwent SASI after SG failure at a tertiary bariatric referral center.
- SASI conversion after SG failure is associated with significant weight loss and favorable metabolic outcomes.
- Mid-term follow-up revealed that 65% of patients experienced resolution of gastroesophageal reflux disease (GERD), although 11.3% developed de novo GERD symptoms.
- Larger, multi-center studies with longer follow-up are needed to further refine the role of SASI in revisional bariatric surgery.

The primary advantages of SASI are linked to its straightforward surgical technique, which involves a single anastomosis between the ileum and the stomach. This approach is not only technically reasonably easy to perform, but also enhances weight loss and metabolic improvements through its malabsorptive effect. A unique feature of SASI is its dual-pathway digestive configuration, where food passes through both the duodenum and directly into the small bowel via the gastroileal anastomosis. This configuration reduces the risk of severe malabsorption while promoting hormonal changes associated with the “ileal brake” effect^[1]^.

Despite the growing interest in SASI, SG remains the most popular bariatric procedure worldwide due to its numerous advantages over more complex procedures involving intestinal bypass. These advantages include unrestricted endoscopic access to the entire digestive tract and the potential for conversion to a variety of procedures, including those with a bypass component, at any time^4^. SASI is particularly appealing in cases where a more pronounced metabolic effect is required or when weight loss results from SG are deemed insufficient.

However, while conversion to SASI may seem like an attractive option for SG failure, several critical issues must be addressed. These include the evolution of gastroesophageal reflux disease (GERD), the risk of protein malnutrition, and the outcomes in terms of weight loss and improvement in obesity-related comorbidities.

The limited availability of long-term follow-up data on SASI remains a critical gap in the current literature and further research with extended follow-up is necessary to fully assess the durability of weight loss, metabolic improvements, and potential late complications. However, mid-term outcomes are essential for refining patient selection criteria and optimizing long-term management strategies for individuals undergoing SASI after SG failure. In this study, we present the results of a single-center series of patients undergoing conversion to SASI for failed SG, with a focus on the mid-term outcomes of this procedure.

## Materials and methods

### Study design

This retrospective study investigated the use of single anastomosis sleeve ileal (SASI) bypass as a second-step procedure following sleeve gastrectomy (SG) failure. Consecutive patients presenting at our tertiary bariatric referral center with SG failure between May 2019 and June 2024 were offered SASI if they met the eligibility criteria. All patients were informed about the available surgical techniques, associated risks, and expected outcomes. Specifically, patients were advised of the experimental nature of SASI especially as a salvage procedure after SG failure. Indeed, SASI is still considered an experimental procedure due to the limited long-term data available on its efficacy, safety, and durability as a revisional bariatric surgery that prevents it from being classified as a standard procedure. Additionally, variations in surgical technique, such as differences in the length of the biliopancreatic limb and the method of anastomosis, contribute to the heterogeneity of reported outcomes.

### Inclusion and exclusion criteria

Inclusion criteria for SASI were:

- SG failure, defined as insufficient weight loss (IWL; <20% total body weight loss) or weight regain (WR; BMI >35 or ≥10% of TWL relative to the nadir weight post-SG)^4^, with or without persistent or recurrent obesity-related comorbidities, including type 2 diabetes (T2D), obstructive sleep apnea syndrome (OSAS), or metabolic syndrome. We selected the 20% TWL threshold for surgical success based on substantial evidence from large observational studies, which have shown that achieving a TWL greater than 20% after bariatric surgery is associated with reduced all-cause mortality, a lower risk of major adverse cardiovascular events, decreased incidence of end-stage renal disease and cancer, as well as an increased likelihood of type 2 diabetes remission^5^–^9^.
- Age between 18 and 60 years.
- Absence of severe esophagitis (≥grade B, see non-inclusion criteria) or Barrett’s esophagus.
- Clearance from a psychiatrist, dietitian, and bariatric surgeon after completing a 6-month preoperative workup and preparation for revisional surgery.

Non-inclusion criteria included:

- Age >60 years.
- Symptomatic gastroesophageal reflux disease (GERD) despite high-dose (≥40 mg per day) proton pump inhibitors (PPIs) or endoscopic evidence of esophagitis grade B or higher (Los Angeles classification), for whom Roux-en-Y gastric bypass (RYGB) was preferred as the revisional procedure.

Exclusion criteria included any intraoperative change in surgical strategy, such as the performance of a bariatric procedure other than SASI due to unplanned intraoperative findings.

### Data collection

Data were prospectively collected and included:

- Demographic and anthropometric data (age, gender, BMI at SG, lowest BMI post-SG, BMI at SASI).
- Comorbidities at the time of SASI.
- Operative details (distance of the gastroileal anastomosis from the ileocecal valve, hiatal hernia repair, re-sleeve of the gastric tube, concomitant procedures, and duration of surgery).
- Short- and mid-term postoperative complications.
- Weight loss and obesity-related comorbidities over time.

All patients underwent preoperative esophagogastroduodenoscopy (EGD) to evaluate gastric tube volume, hiatal hernia, and signs of GERD. Gastric tube volume was further assessed using computed tomography (CT) with 3D reconstruction and gas contrast medium (Duo Gas®). Post-SG stomach dilation was defined as a gastric volume exceeding 250 cc.

### Ethical considerations

Preoperative informed consent was obtained from all patients, ensuring they were fully informed of the procedure, risks, and benefits. The study was approved by the local ethics committee and conducted in compliance with ethical standards and regulatory requirements. The data regarding patients were prospectively collected and recorded on the Italian Society of Bariatric Surgery (SICOB) register, which allows patients’ anonymity and requires access through electronic credentials. The study has been reported in line with the Standards for Quality Improvement Reporting Excellence (SQUIRE) criteria^[5]^, and in line with the 2025 STROCSS guidelines^[6]^.

### Primary and secondary outcomes

Primary outcomes included:

- Weight loss, assessed by changes in BMI and %TWL at 6, 12, 18, and 24 months postoperatively. %TWL was calculated as [(preoperative weight − follow-up weight)/preoperative weight × 100]. For patients lost from follow-up, we took the last weight recorded at last follow-up to avoid potential bias linked to worse results in terms of WL in patients lost from follow-up.
- Improvement or resolution of obesity-related comorbidities.

A subanalysis of weight loss outcomes was conducted based on the patient’s response to SG (IWL or WR) and whether re-sleeve was performed during SASI.

Secondary outcomes included

- Duration of surgery.
- Rates of early (≤30 days) and late (>30 days) postoperative complications, classified according to the Clavien–Dindo classification^[7]^.
- Rates of secondary surgical procedures due to complications related to SASI anatomy.

### Definitions

- T2D: Fasting plasma glucose (FPG) ≥126 mg/dL (7.0 mmol/L) or use of antidiabetic medication. Remission of type 2 diabetes (T2D) was defined as fasting plasma glucose below 126 mg/dL (7.0 mmol/L) without the need for antidiabetic medication, while improvement referred to better glycemic control with reduced medication requirements^[8]^.

- OSAS: Apnea-hypopnea index (AHI) >15 events/hour on polysomnography. Remission was defined as an apnea-hypopnea index (AHI) of fewer than 15 events per hour on polysomnography, whereas improvement was characterized by a significant AHI reduction, with or without continued positive airway pressure therapy^[9]^.

- Metabolic syndrome: Presence of at least three of the following: central obesity (waist circumference ≥94 cm in men, ≥80 cm in women), elevated fasting blood glucose (≥100 mg/dL or 5.6 mmol/L), elevated blood pressure (≥130/85 mmHg or antihypertensive medication), elevated triglycerides (≥150 mg/dL or 1.7 mmol/L), or reduced HDL cholesterol (<40 mg/dL in men, <50 mg/dL in women). Metabolic syndrome was considered in remission when fewer than three diagnostic criteria persisted, while improvement was defined as partial resolution of individual components, including reductions in waist circumference, fasting blood glucose, blood pressure, triglycerides, or increases in HDL cholesterol^[10]^.

### Surgical technique

1. Hiatal hernia repair: The hiatus was assessed for herniation. If present, the hernia was repaired using a posterior approach with careful dissection and closure of the diaphragmatic pillars.
2. Re-sleeve gastrectomy: If significant gastric tube dilation (>250 cc) was identified, a re-sleeve was performed over a 36-Fr bougie. The dilated portion of the gastric tube was resected, and the stomach was restapled to restore the restrictive capacity of the SG.
3. Gastroileal anastomosis: A 250–300 cm ileal loop (depending on BMI) was measured from the ileocecal valve and transposed in an antecolic position^[1-17]^. A side-to-side anastomosis (~3 cm) was created between the ileum and gastric antrum, 3–5 cm proximal to the pylorus, using a 45-mm linear stapler. Given the difference in thickness between the gastric antrum wall and the ileal wall, and in order to find the right balance between the risk of bleeding and leakage, we opted for a purple (medium) reload with Tri-Staple technology (3.0, 3.5, and 4 mm thickness). The anterior wall was closed with a continuous double-layer PDS 2-0 suture. Alternatively, the anastomosis was performed entirely manually. A leak test was performed, and no drain was placed.
4. Postoperative care: Patients were allowed clear fluids on the day of surgery and a soft diet from postoperative day 1. Discharge occurred on the same day.
5. All patients included in the study underwent a comprehensive nutritional assessment, which included systematic blood tests and in-person follow-up.

### Statistical analysis

Categorical variables were described as numbers and percentages, and continuous variables as means ± standard deviations (SDs). Normality was assessed using Shapiro-Wilk tests. Group comparisons were performed using Fisher’s exact test (for 2 × 2 tables) or the chi-squared test (for larger tables) for categorical variables and the Mann-Whitney U test for continuous variables. All tests were two-tailed, with a significance level of *P* <0.05.

Logistic regression models were built to assess predictors of postoperative outcomes (GERD, diabetes mellitus type 2 [T2D], suboptimal weight loss (%TWL at 2 years <20%) following bariatric surgery. Variables included age, sex, baseline BMI (at sleeve and SASI), minimum postoperative weight, percent total weight loss (%TWL) at 2 years, preoperative comorbidities (GERD, T2D, banding status), hiatal hernia repair. Continuous variables were retained as linear terms, and categorical variables (e.g., sex, banding status, hiatal hernia repair) were coded as binary (0/1). Missing data were addressed via listwise exclusion, retaining only complete cases for each model. Model assumptions were verified, including multicollinearity (variance inflation factor [VIF] <5 for all predictors) and linearity of the logit. Odds ratios (ORs), 95% confidence intervals (CIs), and *P* values were calculated using maximum likelihood estimation. Model performance was evaluated using Nagelkerke’s pseudo-R^2^ and the Akaike Information Criterion (AIC). Analyses were conducted in R Statistical Software (version 4.3.1), and significance was set at *P* <0.05.

## Results

**Figure 1.**
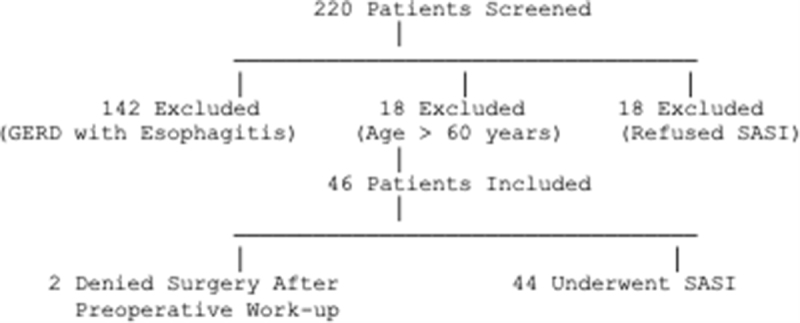
Flowchart of patient selection and outcomes. *Abbreviations*: GERD, gastroesophageal reflux disease; SASI, single anastomosis sleeve ileal bypass.

A total of 220 consecutive patients were screened, and 44 fulfilled the inclusion criteria for the present study (Fig. 1). Preoperative characteristics are summarized in Table 1. The majority of patients were female (86.3%) with a mean age at the time of SASI of 42.6 ± 13 years. Additionally, 8 patients (18.2%) had a history of gastric banding before LSG.

**Table 1:** Demographic and clinical characteristics of patients undergoing conversion from SG to SASI (n = 44)

Variable	Value
Age (mean ± SD), years	42.6 ± 13
Gender (M/F)	6/38 (13.6%)
History of gastric banding, n (%)	8 (18.2%)
BMI at SG (mean ± SD), kg/m^2^	47.9 ± 10.2
BMI at SASI (mean ± SD), kg/m^2^	39.1 ± 7.2
Indication for SASI, n (%)	
-Insufficient weight loss (IWL)	20 (45.4%)
-Weight regain (WR)	24 (55.6%)
Weight loss with comorbidities, n (%)	
-OSAS	7 (15.9%)
-T2D	3(6.8%)
-HT	6 (13.6%)
-Metabolic syndrome	5 (11.3%)

HT, hypertension; IWL, insufficient weight loss; Met Syn, metabolic syndrome; OSAS, obstructive sleep apnea syndrome; T2D, type 2 diabetes; WR, weight regain.

The mean BMI at the time of index SG was 47.9 ± 10.2 kg/m^2^ and 39.1 ± 7.2 kg/m^2^ at the time of conversion to SASI. The mean interval between the two procedures was 75.1 ± 38.7 months. The indications for conversion of SG to SASI were failure due to insufficient weight loss in 20 patients (45.5%) and weight regain in 24 patients (54.5%). Twenty patients (45.5%) presented with gastroesophageal reflux disease (GERD), and 10 of these (50%) exhibited signs of grade A esophagitis on preoperative endoscopy. Among these patients, 10 were taking 20 mg of PPIs two to three times monthly, 6 were taking 20 mg of PPIs one or two times weekly, and 4 were taking 20 mg on alternate days. Five of these patients with GERD had a history of gastric banding prior to LSG. A hiatal hernia was preoperatively diagnosed in 9 patients through EGDS and CT. In 4 of these patients, it measured approximately 2 cm, in another 4 it measured approximately 3 cm, and in one patient, the hernia was larger than 4 cm. All the patients had GERD symptoms. Regarding comorbidities, 5 patients (11.3%) had metabolic syndrome, 3 (6.8%) had type 2 diabetes, 7 (15.9%) had obstructive sleep apnea syndrome (OSAS) treated with CPAP, and 6 (13.6%) had hypertension (Table 1).

### Intraoperative and immediate postoperative results

The mean duration of surgery was 66 ± 15.5 minutes (range: 45–100 minutes). A total of 35 associated procedures were performed in 27 patients (61.3%), most commonly (81.5% of cases) the recalibration of the SG (Re-SG) over a 36 Fr endoluminal bougie. In two cases, the gastric tube was reduced in volume using an oversewn inverting running suture. Posterior hiatal hernia repair was conducted in 9 patients, of whom 8 also underwent a concurrent Re-SG. Additionally, two patients had an associated cholecystectomy for symptomatic gallbladder stones. There was no postoperative mortality and no laparotomy conversion. The overall short-term complication rate (<30 days) was 11.3% (n = 5). Four patients experienced intraluminal bleeding at the gastro-ileal anastomosis, which was managed conservatively (Clavien–Dindo Grade 2). One patient undergoing resleeve developed a leak at the upper third of the stapled line on postoperative day 8 (Clavien–Dindo Grade 3b). This patient, admitted in septic shock, underwent laparoscopic reoperation, extensive abdominal lavage, and fistula drainage, followed by endoscopic placement of a pigtail drain, achieving full recovery by postoperative day 36 (Table 2).

**Table 2:** Results of conversion from SG to SASI

Variable	Value
Duration of surgery (mean ± SD), min	66 ± 15.5
Associated procedures, n	35
- Re-SG	22
- SG tube oversewing	2
- Hiatal hernia (HH) repair	9
- Cholecystectomy	2
Immediate postoperative complications, n (%)	
- Bleeding from gastroenterostomy	4 (9.1%)
- Anastomotic leak	1 (2.2%)
Long-term complications, n (%)	
- De novo GERD	5 (11.4%)
- Occasional diarrhea	5(11.4%)
- Hypoalbuminemia	2 (4.5%)
- Low iron level	2 (4.5%)
- Severe malnutrition	1 (2.2%)
Surgeries for SASI-related complications, n	
- Conversion to RYGB for GERD	1
- Conversion to SG for excessive weight loss	1

GERD, gastroesophageal reflux disease; HH, hiatal hernia; Re-SG, re-sleeve gastrectomy; RYGB, Roux-en-Y gastric bypass; SG, sleeve gastrectomy; WL, weight loss.

### Primary outcome

Table 3 presents weight loss results after conversion to SASI. The mean follow-up duration was 33.8 ± 17.4 months, with data available for 41 and 28 patients at 1 and 2 years postoperatively, respectively. At 12 and 24 months, the mean BMI decreased to 30.5 ± 5.9 kg/m^2^ and 27.5 ± 4.8 kg/m^2^, respectively. The percentage of total weight loss (%TWL) at 12 and 24 months was 21.5 ± 7.8% and 29.7 ± 9.5%, respectively. Analysis of the resleeve versus non-resleeve subgroups showed that although greater weight loss was observed in the resleeve group, the differences at 1 and 2 years were not statistically significant (*P* = 0.81 and *P* = 0.49, respectively). Similarly, no statistically significant differences were observed between WR and IWR at 1 and 2 years (*P* = 0.83 and *P* = 0.74, respectively) (Table 4). Patients with a previous history of gastric banding demonstrated a weight loss response comparable to that of other study participants. Specifically, at 1 year, the % TWL was 21.9 ± 8.8, and at 2 years (7 out of 8 available patients), it was 32.9 ± 7.

**Table 3:** weight loss results after SASI

	N eligible pts	N present pts	% of FU	BMI	%TWL
6 po months	44	44	100%	33.5 ± 6.5	14.1 ± 6.9
12 po months	42	41	97.6%	30.5 ± 5.9	21.5 ± 7.7
18 po months	39	33	84.6%	29.1 ± 5.9	25.9 ± 8.9
24 po months	32	28	87.5%	27.5 ± 4.8	29.7 ± 9.5

BMI: body mass index; FU: follow-up; po: postoperative; pts: patients; %TWL: percentage total weight loss.

**Table 4:** Comparison of weight loss results in different groups of patients after SASI

	WR	n	IWL	n	*P*	RE-sleeve	*n*	Not re-sleeve	*n*	*P*
BMI at 1 po y.	28.8 ± 4.2	21	32.3 ± 7	20	0.28	30.7 ± 5.4	24	30.2 ± 6.8	17	0.81
BMI at 2 po y.	26.6 ± 4.2	15	28.6 ± 5.4	13	0.71	27.8 ± 5.2	19	27 ± 3.9	9	0.23
%TWL at 1 po y.	22.1 ± 6.9	21	20.9 ± 8.4	20	0.83	22.9 ± 8.2	24	19. 6 ± 6.8	17	0.81
%TWL at 2 po y.	29.5 ± 4.7	14	29.3 ± 10.6	13	0.74	32.5 ± 9.4	19	23.6 ± 7	9	0.49

BMI: body mass index expressed in Kg/m^2^; %EWL: percentage excess weight loss; IWL: insufficient weight loss; po: postoperative; %TWL: percentage total weight loss; WR: weight regain; y: years.

### Mid-term results

Mid-term complications following SASI conversion were predominantly associated with GERD. Thirteen of 20 patients (65%) experienced GERD symptom resolution, while 7 patients (35%) required continued PPI therapy despite prior hiatal hernia repair and gastroenterostomy. Additionally, de novo GERD necessitating PPI treatment was reported in 5 patients (11.3%) during follow-up. One patient experienced severe, invalidating GERD symptoms refractory to PPI therapy, necessitating conversion to a Roux-en-Y gastric bypass (RYGB), which resolved the GERD.

The most serious complication observed in our study was significant weight loss and severe protein malnutrition, which occurred in one patient. This patient had a highly advanced metabolic profile, meeting all five criteria for metabolic syndrome, along with severe obstructive sleep apnea syndrome (SAOS) and poorly controlled type 2 diabetes (T2D). Despite an initial BMI of 40, the decision was made to convert SG to SASI due to the severity of metabolic disease. Although the patient experienced remarkable improvement in comorbidities postoperatively, progressive protein malnutrition ensued, initially managed with enteral feeding but ultimately requiring parenteral nutrition. Reversal to the original SG anatomy resolved the malnutrition but was followed by rapid recurrence of metabolic comorbidities, as anticipated.

Five patients (11.3%) experienced intermittent diarrhea, managed conservatively without nutritional deficiencies. Two patients (4.5%) developed mild hypoalbuminemia, which resolved with oral protein supplementation, and 2 (4.5%) had iron deficiency with low ferritin levels, treated with oral iron without hospitalization (Table 2).

### Comorbidity resolution

Comorbidities responded well to SASI. Among the 7 patients with hypertension, 4 (57.4%) reduced their medication dosage, and 3 (42.8%) discontinued medications. Diabetes resolved completely in 2 patients (66.6%) and stabilized in the remaining patient with a reduced dosage of hypoglycemic agents. All patients with severe OSAS discontinued CPAP therapy. Regarding patients with metabolic syndrome, we recorded an improvement in two patients and a complete resolution in three. All showed a significant reduction in abdominal circumference, normalization of blood glucose levels, and stabilization of blood pressure values.

### Comorbidity multivariate logistic regression analysis

Multivariate logistic regression identified preoperative BMI at SG (OR = 1.13, 95% CI = 1.02–1.25, *P* = 0.02), minimum postoperative BMI after SG (OR = 0.84, 95% CI = 0.73–0.96, *P* = 0.01), and banding (OR = 3.32, 95% CI = 1.05–10.50, *P* = 0.04) as independent predictors of suboptimal weight loss (%TWL <20% at 2 years). Higher preoperative BMI increased the likelihood of inadequate weight loss, whereas achieving a lower postoperative BMI after SG was protective. Patients with previous history of gastric banding had over threefold higher odds of suboptimal outcomes. Age, sex, and BMI at SASI did not significantly correlate with poor weight loss (*P* > 0.05). The model demonstrated good discrimination (AUC = 0.78), highlighting the importance of preoperative optimization and cautious use of SASI in individuals with previous history of gastric banding (Table 5).

**Table 5:** Multivariate logistic regression analysis for TWL less than 20% at 2 years after SASI

Variable	Odds ratio (OR)	95% CI	*P* value
Age	1.02	[0.97–1.08]	0.40
Sex (male)	0.64	[0.26–1.56]	0.32
BMI at sleeve	1.13	[1.02–1.25]	**0.02**
Minimum BMI after SG	0.84	[0.73–0.96]	**0.01**
BMI at SASI	1.05	[0.97–1.14]	0.25
GB	3.32	[1.05–10.50]	**0.04**

BMI: body mass index; GB: gastric banding; SASI: single anastomosis sleeve ileal bypass, SG: sleeve gastrectomy. Statistically significant values are highlighted in bold.

Multivariate logistic regression identified previous history of gastric banding (OR = 6.05, 95% CI = 1.85–19.80, *P* = 0.003) and preoperative GERD (OR = 12.18, 95% CI = 3.45–43.10, *P* < 0.001) as significant independent predictors of postoperative GERD following SASI bypass. Greater % TWL at 2 years (%TWL) reduced GERD risk (OR = 0.89, 95% CI = 0.80–0.99, *P* = 0.04). Age, sex, BMI at SG, BMI at SASI and hiatal hernia repair did not independently correlate with GERD postop. The model demonstrated excellent discrimination (AUC = 0.85), underscoring the critical role of preoperative GERD status and banding avoidance in mitigating postoperative reflux complications (Table 6).

**Table 6:** Multivariate logistic regression analysis for the presence of GERD at 2 years after SASI

Variable	Odds ratio (OR)	95% confidence interval (CI)	*P* value
Age	1.01	[0.95–1.08]	0.75
Sex (male)	0.74	[0.28–1.97]	0.55
BMI at sleeve	1.05	[0.97–1.14]	0.22
Minimum BMI postoperative	0.90	[0.80–1.02]	0.08
BMI at SASI	1.03	[0.95–1.12]	0.45
%TWL at 2 years	0.89	[0.80–0.99]	**0.04**
GB	6.05	[1.85–19.80]	**0.003**
Preoperative GERD	12.18	[3.45–43.10]	**<0.001**

BMI: body mass index; GB: gastric banding, GERD: gastro-esophageal reflux disease; SASI: single anastomosis sleeve ileal bypass; %TWL: percentage total weight loss. Statistically significant values are highlighted in bold.

Multivariate logistic regression model for postoperative T2D identified two significant predictors. Greater total weight loss at 2 years (%TWL) (adjusted OR = 0.88 per 1% increase, 95% CI: 0.78–0.99, *P* = 0.04) and preoperative T2D (adjusted OR = 12.5, 95% CI: 2.50–62.5, *P* = 0.003) predicted postoperative T2D. Baseline BMI at SG trended toward significance (OR = 1.12, *P* = 0.07), suggesting a potential role for preoperative obesity severity. Age, sex, postoperative minimum weight, and BMI at SASI showed no significant associations (*P* > 0.05). These findings underscore the importance of sustained weight loss and preoperative diabetes status in predicting postoperative glycemic outcomes, warranting validation in larger cohorts.

## Discussion

This study demonstrates that the conversion of a failed sleeve gastrectomy (SG) to single anastomosis sleeve ileal (SASI) bypass due to insufficient weight loss or weight regain yields favorable outcomes, with a %TWL exceeding 20% at both 1 and 2 years postoperatively. Furthermore, the procedure exhibits a strong antimetabolic effect, leading to remission of most metabolic comorbidities within our patient cohort. Our findings align with existing literature, though studies evaluating SASI, particularly as a revisional procedure, remain limited and are often constrained by short follow-up periods.

In a multicenter study by Mahdy *et al*, which included 551 patients, a subset of 58 underwent SASI as a salvage procedure following failed SG. The authors reported a complete remission of diabetes and a %TWL of 17.3% at one year^[18]^. Similarly, Hassan *et al* analyzed 50 patients who underwent revisional SASI for weight regain after SG and found an excess weight loss percentage (EWL%) of 85% at one year, again demonstrating excellent diabetes control^[19]^. These impressive metabolic effects appear to be related to the hormonal stimulation of the distal intestine following gastro-ileal anastomosis, a process known as digestive adaptation^[20]^. This adaptation involves reduced nutrient absorption in the proximal intestine and increased absorption at the distal ileum, leading to hypertrophy of ileal L cells and increased secretion of gut hormones, including glucagon-like peptide 1 (GLP-1), peptide YY (PYY), oxyntomodulin (OXM), and fibroblast growth factor 19 (FGF-19). Notably, elevated GLP-1 levels promote insulin secretion, enhance glucose and lipid clearance, slow gastric emptying, and induce a profound sense of satiety – referred to as “functional satiety” – which surpasses the mechanical satiety achieved through gastric restriction alone^[21–23]^.

Several salvage techniques have been proposed following the failure of SG, whether due to weight regain (WR) or insufficient weight loss (IWL)^[24]^. In particular, the addition of a malabsorptive component appears to be essential in achieving better outcomes. Andalib *et al*, in a comparison of resleeve, Roux-en-Y gastric bypass (RYGB), biliopancreatic diversion with duodenal switch (BPD/DS), and single anastomosis duodenal switch (SADS), reported significantly higher %TWL (between 10% and 14%) in procedures incorporating malabsorption compared to resleeve, at just over 1 year of follow-up^[25]^. However, as expected, the rates of both early and late complications were also significantly higher. Similar results were observed by Homan *et al* in a comparative analysis between RYGB and BPD/DS at 34 months post-LSG conversion^[26]^. Again, although BPD/DS yielded better weight loss outcomes, it also showed a higher rate of short-term complications and vitamin deficiencies. In a multicenter study on a large patient cohort, Rayman *et al* found that one anastomosis gastric bypass (OAGB) resulted in better weight loss than RYGB at 29 months post-conversion (23% vs. 16%). However, OAGB was associated with a significantly higher rate of GERD (17.4% vs. 7.6%)^[27]^. Regarding SASI as a salvage procedure after LSG, the available literature is extremely scarce, and no comparative studies exist between SASI and other techniques. However, the weight loss outcomes and postoperative complication rates observed in this study are fully comparable to those of other procedures and, in some cases, even superior. Moreover, compared to the aforementioned techniques, SASI offers several undeniable advantages, such as easy reversibility in cases of excessive weight loss or malnutrition, a lower risk of internal hernia, and anastomotic ulcer due to the rich vascularization of the antrum and reduced tension on the anastomosis. Moreover, SASI preserves pyloric function and provides dual pathways for nutrient flow. This anatomical advantage may reduce the risk of dumping syndrome, enhance nutrient absorption, and maintain incretin stimulation to counteract metabolic disturbances. Nevertheless, close and systematic monitoring of nutritional parameters is essential to prevent complications such as protein malnutrition and vitamin deficiencies. Regular assessment of protein levels, micronutrients (especially iron, calcium, vitamin D, and B12), and overall nutritional status is mandatory. Early detection and timely intervention with appropriate supplementation and dietary counseling are critical to managing these risks and avoid long-term nutritional complications.

The primary immediate postoperative complication in our study was bleeding at the gastroenterostomy site. However, this was successfully managed endoscopically in all cases, with only one patient requiring a blood transfusion. The challenge arises from the use of mechanical stapling to create the anastomosis between the stomach and ileum, as these tissues exhibit varying thicknesses, complicating the selection of an appropriate stapler load. While a similar issue is encountered with the gastrojejunostomy in Roux-en-Y gastric bypass, the stomach is generally thicker at the antral region where the SASI gastroenterostomy is performed. One potential solution is to construct the anastomosis using a hand-sewn technique, while another involves meticulous intraoperative hemostatic control. To mitigate the risk of bleeding, we now request anesthesiologists to transiently increase blood pressure and verify hemostasis within the anastomosis lumen before completing the anterior closure, effectively resolving this complication.

GERD remains the Achilles’ heel of SG and is a crucial factor when considering revisional procedures. While GERD after SG is multifactorial and complex, intrathoracic migration of the gastric tube is regarded as a primary contributor^[23,28,29]^. Therefore, we strongly advocate for a thorough assessment of the esophageal hiatus at the outset of the procedure, with hiatal hernia repair performed when necessary. A posterior approach is essential for full exposure of the hiatus, which is then closed by approximating the crural pillars. Additionally, Hutopila *et al* recently demonstrated excellent outcomes with esophageal fixation to the crura, preventing superior migration of the gastric tube – an important technical consideration in revisional SG cases^[29]^. We found that a previous history of gastric banding and the presence of preoperative GERD were independently associated with postoperative GERD while greater weight loss has a protective effect. These data may reflect the difficulty in addressing the hiatal hernia in individuals with a history of banding. For patients with significant pre-existing GERD or hiatal hernia, Roux-en-Y gastric bypass may represent a more appropriate revisional option, as illustrated by the one patient in our cohort who required conversion to RYGB to achieve GERD resolution.

SASI as a primary procedure has been shown to be more effective than SG in controlling GERD^[30–32]^. In our study, despite the anatomical alterations already induced by SG, SASI resulted in significant GERD improvement. This may be attributed to the gastro-ileal anastomosis, which lowers intragastric pressure and accelerates gastric emptying, thereby altering the flow dynamics of gastric contents^[2,31]^. However, our findings also indicate that de novo GERD developed in approximately 11% of cases, suggesting that the omega loop anatomy, which allows intestinal secretions to enter the stomach, may contribute to GERD symptoms.

Another technical consideration is the resizing of the gastric tube when it is excessively dilated, either due to physiological adaptation or inadequate calibration during the primary SG^[33]^. After dissecting the hiatus, the gastric tube can be reliably assessed for dilation and reshaped accordingly. Indeed, several studies have shown that postoperative BMI can be influenced by the volume of the remaining gastric tube. For this reason, all patients underwent gastric volumetry via CT scan to determine whether resizing of the gastric pouch was necessary during the SASI bypass procedure. In the present study, a sleeve was considered dilated if its volume exceeded 250 cc. This cut-off was derived from previous studies in the literature. In fact, while the optimal gastric capacity after sleeve gastrectomy is considered to be 100–120 ml, Braghetto *et al* reported that the average gastric volume at 2 years post-surgery is already around 250 ml^[34,35]^. Similarly, Disse *et al* found that in patients with sleeve dilation, the gastric tube volume increased from 127 ml at 3 months post-surgery to 245 ml at 1 year^[36]^. Although various factors can contribute to the failure of a restrictive technique such as LSG, the average gastric volume in patients experiencing weight regain (WR) increases from 120 ml immediately after surgery to over 500 ml at 5 years post-LSG^[37]^. However, the addition of a new staple line increases the risk of bleeding and leaks, particularly in patients with a history of gastric banding^[38]^. In such cases, an alternative approach involves plicating the gastric tube using an inverting running suture, which provides additional restriction without the risks associated with stapling.

Although SASI has a malabsorptive component, the risk of nutritional deficiencies and malnutrition appears to be lower compared to other malabsorptive procedures. Nevertheless, one patient in our cohort developed severe malnutrition that necessitated surgical reversal to restore the original sleeve anatomy. This case underscores the importance of emphasizing to patients the need for long-term follow-up and strict adherence to postoperative supplementation with vitamins and protein. While the technical reversal of SASI to a sleeve gastrectomy is relatively straightforward, it unfortunately results in the recurrence of metabolic comorbidities such as type 2 diabetes and metabolic syndrome, as well as weight gain.

This study has four primary limitations: a small sample size, a relatively short follow-up period, the lack of quality of life evaluation, and its retrospective nature. The retrospective nature may indeed introduce biases, such as selection bias, and the small sample size limits the statistical power, potentially affecting the generalizability and robustness of the results. All procedures in the present study were performed at a high-volume tertiary center, which may limit generalizability to smaller institutions with different levels of surgical expertise. These factors should be considered when interpreting our findings that make it difficult to draw definitive conclusions regarding the long-term efficacy and safety of SASI. Given the relatively novel nature of SASI, it is not yet widely adopted, which makes large-scale, nationwide administrative studies challenging at this time. To address these limitations, prospective, multicenter studies with larger cohorts and longer follow-up periods are needed to provide more robust and generalizable data on the long-term outcomes of SASI as a revisional procedure for SG failure.

Moreover, a longer follow-up would provide more robust evidence regarding the long-term efficacy of SASI as a salvage procedure for failed SG. Nevertheless, our follow-up duration remains among the longest currently reported in the literature for this technique. Despite these limitations, our study is the first in the literature to specifically report data on SASI in the setting of SG failure. Previous series have included mixed cohorts of primary and revisional SASI cases, which limits the interpretability of their results^[18]^. By focusing solely on SG failure, our study provides clearer insights into the outcomes of SASI as a revisional procedure.

Finally, quality of life is an essential matter when we discuss bariatric surgery results, especially concerning a malabsorptive technique. The lack of formal quality of life assessment tools (e.g., BAROS, SF-36) is a limitation of this study, and future research should include standardized patient-reported outcomes

## Conclusion

SASI is an intriguing niche procedure in metabolic and bariatric surgery, offering a powerful hybrid antimetabolic effect while maintaining the advantage of being readily reversible if needed. While the present study provides valuable mid-term outcomes, future studies should evaluate the long-term durability of SASI, particularly in comparison to other revisional techniques such as RYGB, with extended follow-up beyond 5 years. Future research is mandatory to better understand the long-term efficacy and safety of SASI.

## Data Availability

Availability of data: Data collected for the study, including deidentified participant data and a data dictionary defining each field, will be made available to authorized researchers upon request. Data sharing will comply with ethical guidelines for medical research. Types of data available: Researchers may access deidentified participant data and the data dictionary defining variables used in the study. No directly identifiable information (e.g., names, social security numbers) will be shared. Additional data sets may be available upon request, subject to regulatory approval. Additional documents: Depending on the study, related documents such as the study protocol, statistical analysis plan, and informed consent form may be made available upon request. Access location: Data will be shared via a secure online repository, institutional servers, or through direct request to the principal investigator. The specific access link or email contact will be provided upon request. Access criteria: Data will be shared only with qualified researchers affiliated with recognized institutions. Researchers must submit a formal proposal detailing the intended analyses. Access is subject to approval by an ethics committee and/or CNIL. A data access agreement (DAA) must be signed, ensuring compliance with GDPR, data security, and confidentiality rules.

## Research registration unique identifying number (UIN)

In accordance with the Declaration of Helsinki, the present study was registered with the number researchregistry11281. This research study was retrospectively registered.
